# Integrated Single-Cell Analysis Dissects Regulatory Mechanisms Underlying Tumor-Associated Macrophage Plasticity in Hepatocellular Carcinoma

**DOI:** 10.3390/genes16070817

**Published:** 2025-07-12

**Authors:** Yu Gu, Wenyong Zhu, Zhihui Zhang, Huiling Shu, Hao Huang, Xiao Sun

**Affiliations:** 1 State Key Laboratory of Digital Medical Engineering, School of Biological Science and Medical Engineering, Southeast University, Nanjing 210096, China; 2 College of Acupuncture-Moxibustion and Tuina, Nanjing University of Chinese Medicine, Nanjing 210023, China

**Keywords:** hepatocellular carcinoma, single-cell omics, tumor-associated macrophages, gene regulation, biomarkers

## Abstract

**Background**: Tumor-associated macrophages (TAMs) are critical regulators of the hepatocellular carcinoma (HCC) microenvironment, yet their epigenetic heterogeneity and regulatory programs remain poorly defined. **Methods**: We performed integrative analysis on single-cell RNA-seq and ATAC-seq profiling of HCC patients to dissect TAM subtypes at high resolution. By correlating chromatin accessibility with gene expression, we identified cell-type-specific candidate *cis*-regulatory elements (CREs). TAM subsets with prognostic significance were determined through integration with HCC clinical cohorts. Pseudotime and multi-regional analyses were used to uncover regulatory trajectories underlying macrophage phenotypic transitions. The identification framework of a super-enhancer (SE) was constructed, and potential therapeutic targets were prioritized using drug–gene interaction data. **Results**: We delineated the regulatory landscape of TAMs in HCC, revealing cell-type-specific chromatin accessibility patterns underlying TAM heterogeneity. The 65,342 CREs linked to gene expression were identified, with distal CREs contributing most to cell-type-specific regulation. Notably, SPP1^+^ TAMs were found to be enriched in tumor cores and associated with poor prognosis in HCC. Liver-resident Kupffer cells showed progressive loss of the core transcription factors SPIC and MAFB, suggesting a potential transition into SPP1^+^ TAMs under tumor pressure. We identified 133 SPP1^+^ TAM-specific SEs and constructed a TF–SE–target gene regulatory network. Notably, 13 target genes showed higher drug–gene interaction effects, highlighting their therapeutic potential. **Conclusions**: This study provides the chromatin accessibility map of TAMs in HCC and reveals how distal CRE-driven transcriptional programs shape TAM states. Our findings lay the foundation for understanding the epigenetic regulation of TAM heterogeneity and nominate potential targets for TAM-directed immunotherapy in HCC.

## 1. Introduction

Hepatocellular carcinoma (HCC), the predominant subtype of primary liver cancer, constitutes the fourth leading cause of cancer-related deaths worldwide [1]. One of the key features of HCC is the pronounced tumor heterogeneity [2]. The tumor microenvironment (TME) exerts a central role in shaping the heterogeneity, and its cellular composition exerts influence on tumor initiation, progression, and therapeutic response [3,4]. Notably, clinical and experimental evidence has indicated a high level of tumor-associated macrophage (TAM) infiltration in the TME in HCC [5,6]. TAMs tend to acquire immunosuppressive and tumor-supportive features, thereby contributing to tumor progression and poor outcomes, emerging as important targets for HCC therapy [7,8].

TAMs represent a highly plastic population [9]. Based on in vitro models, the traditional paradigm classifies macrophages into M1 and M2 phenotypes [10,11]. However, accumulating evidence highlights the limitations of this binary framework. Some TAM subsets simultaneously exhibit or fail to express genes associated with the M1 and M2 phenotypes [12,13]. Therefore, a deep understanding of TAM heterogeneity is urgently needed. Recent advances in high-resolution profiling techniques, such as single-cell RNA sequencing (scRNA-seq) and single-cell ATAC sequencing (scATAC-seq), have expanded the understanding of the cellular composition and gene expression characteristics in the TME [14,15]. scRNA-seq provides a detailed transcriptomic map, enabling the identification of diverse subpopulations based on expression profiles [16]. In various cancers, including HCC, the functional heterogeneity of TAMs has been revealed [17,18,19]. Defining critical TAM subpopulations has become a major strategy for reprogramming pro-tumorigenic into anti-tumor states, representing a promising direction in TAM-targeted therapy.

Although previous studies have advanced our understanding, increasing attention has been directed towards the non-coding regions of the genome [20,21]. The functional cell state is determined by a transcriptional program, which is stringently regulated by the underlying epigenetic landscape, including chromatin accessibility and the activity of *cis*-regulatory elements (CREs), such as promoters and enhancers [22,23,24,25]. Analyzing chromatin accessibility in different cell types or disease states can help us gain a better understanding of molecular programs. Furthermore, the binding and activation of transcription factors (TFs) at specific CREs constitute a major regulatory force governing cellular state transitions [26,27]. Epigenetic profiling, particularly through scATAC-seq, enables the identification of accessible chromatin regions and the detection of CREs and TF binding sites that precede and control gene activation [28,29]. These include super-enhancers (SEs), which are large clusters of regulatory elements characterized by high TF occupancy and active chromatin marks [30,31]. These elements act as central nodes within gene regulatory networks and are increasingly recognized as key determinants of cell identity and disease progression [32,33]. scATAC-seq and scRNA-seq are often used as complementary technologies, delivering a comprehensive picture of the cell identity that integrates transcriptome and transcriptional regulation. Recent studies integrating scRNA-seq and scATAC-seq have defined epigenetic cues underlying the heterogeneity of non-malignant cells in the TME and have highlighted the therapeutic relevance of enhancer and SE regions [34,35,36]. However, in HCC, a generalized description of chromatin states and landscapes of regulatory elements in TAMs is still lacking.

**Figure 1:**
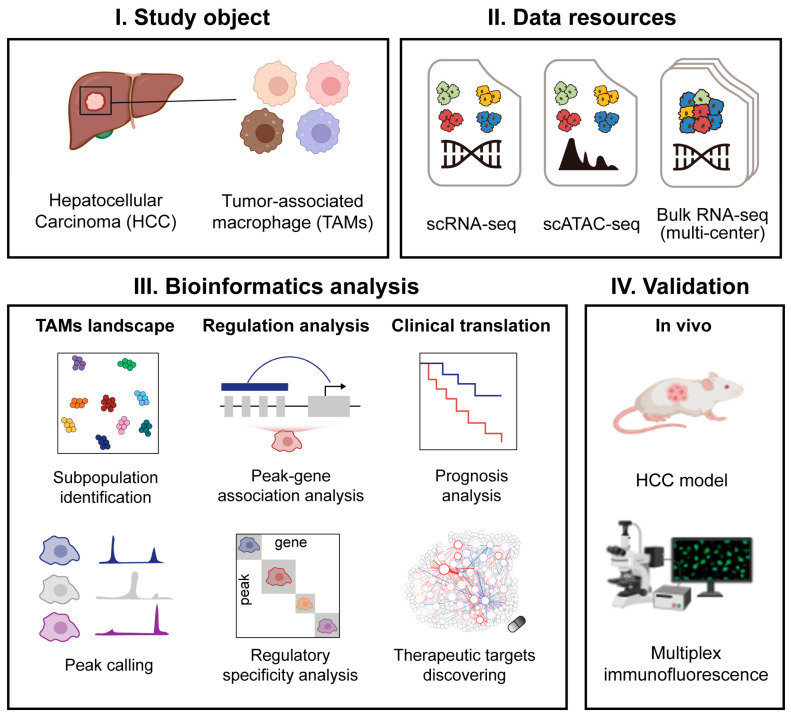
Study design.

In this study, we performed integrative analyses of scRNA-seq and scATAC-seq data on HCC patients to systematically delineate the regulatory landscape of TAMs. We identified key macrophage subtypes with distinct clinical relevance and uncovered a transcriptional reprogramming trajectory from liver-resident Kupffer cells to pro-tumorigenic SPP1^+^ TAMs. Furthermore, by establishing a super-enhancer identification framework and incorporating drug–gene interaction analysis, we uncovered potential therapeutic targets specific to the SPP1^+^ TAM subtype. Together, our findings provide important insights into the epigenetic regulation of macrophage heterogeneity in HCC and lay the groundwork for the development of macrophage-targeted interventions (Figure 1).

## 2. Materials and Methods

### 2.1. Data Acquisition

In this study, we performed integrative analyses of scATAC-seq, scRNA-seq, and bulk RNA-seq datasets from HCC patients (Table S1). The scATAC-seq data were obtained from the NCBI BioProject (accession number: PRJNA944258), consisting of tumor tissue samples from 12 patients. The paired scRNA-seq data for the same cohort were retrieved from the Gene Expression Omnibus (GEO) under accession numbers GSE151530 and GSE189903. To investigate the spatial heterogeneity of macrophage subtypes and gene expression, we further included multi-regional scRNA-seq datasets from GEO (GSE156337, GSE140228, and GSE189903), in which liver samples were collected from non-tumoral tissue, peri-tumoral regions, and tumor cores of the same patients. For clinical validation of macrophage subtypes and target gene expression, we analyzed bulk RNA-seq data from four independent HCC cohorts with prognostic information: the TCGA-LIHC cohort from the TCGA data portal (http://gdac.broadinstitute.org/, accessed on 20 September 2024), the LIRI-JP cohort from the International Cancer Genome Consortium (ICGC) portal (https://dcc.icgc.org/, accessed on 20 September 2024), and two GEO microarray datasets (GSE14520 and GSE116174).

### 2.2. Data Processing

For scRNA-seq data, the pre-processed count matrices were obtained, and further quality control was conducted using the Seurat package (v4.3.0) in R software. Cells were excluded if they expressed fewer than 200 genes or had over 20% mitochondrial RNA content. Doublets were detected and removed using DoubletFinder. Data normalization was performed using the NormalizeData function. Principal component analysis (PCA) was then applied for dimensionality reduction. Based on the Euclidean distance in PCA space, we constructed a k-nearest neighbor graph and selected the top 30 principal components for clustering. Cell clusters were identified using the Louvain algorithm with a resolution of 0.5, and the results were visualized using Uniform Manifold Approximation and Projection (UMAP). We used the FindIntegrationAnchors and IntegrateData functions to correct for batch effects across different patient samples. All data were log-normalized, and highly variable genes were used for anchor selection.

For scATAC-seq data, raw reads were processed using the Cell Ranger ATAC pipeline (v2.1.0, 10× Genomics) and aligned to the GRCh38 reference genome to generate chromatin accessibility peak matrices. Subsequent analyses were performed using the ArchR (v1.0.3) [37]. Only high-quality individual cells were kept for downstream analysis (doublet filter ratio = 2.0, minimum fragments per cell = 1000, minimum Transcription Start Site (TSS) enrichment score = 4). We converted the fragments file into a 500 bp tile matrix of fragment counts using the addTileMatrix function. Dimensionality reduction was performed on the TileMatrix using latent semantic indexing (LSI) and retaining the top 30 components. Clustering was conducted with a resolution of 0.5, and UMAP was used for the visualization of the low-dimensional embedding. We used the Harmony integration to incorporate batch effect correction in the ArchR framework, which ensures alignment across batches and donors.

To integrate scRNA-seq and scATAC-seq data, we used the addGeneIntegrationMatrix function in ArchR, which aligns gene activity scores from scATAC-seq to the scRNA-seq-defined transcriptomic space. This method accounts for modality-specific scaling and includes an integration score that allows quality filtering.

### 2.3. Subpopulation Annotation in TAMs

In scRNA-seq data, clusters were manually annotated based on the expression of canonical marker genes. Hepatocytes were identified by the expression of *HP* and *KRT8*, endothelial cells by *ACTA2* and *RGS5*, and fibroblasts by *GNG11* and *VWF*. Immune cell populations were further classified into macrophages (*CD163* and *CD68*), dendritic cells (*CD1E* and *CD1C*), natural killer (NK) cells (*NKG7* and *KLRF1*), B cells (*CD79A* and *MZB1*), and T cells (*CD3D* and *CD3E*). For scATAC-seq data, gene activity scores were calculated using the addGeneScoreMatrix function, which inferred gene expression from chromatin accessibility. To address the sparsity of scATAC-seq data, we applied the Markov Affinity-based Graph Imputation of Cells (MAGIC, v2.0) to smooth gene activity scores [38]. Major cell types were then annotated based on the scores around the same markers used for scRNA-seq annotation.

We subset the TAMs and employed a two-step approach for the annotation. Firstly, subtype-specific marker genes were primarily curated from previously published studies on TAMs in HCC [39]. These literature-derived markers were complemented by the de novo identification of differentially expressed genes (DEGs) from our scRNA-seq dataset using the FindAllMarkers function in Seurat (avg_log2FC > 0.5 and *p*-value < 0.05). The GO enrichment analysis for DEGs was performed using the enrichGO function from the clusterProfiler package for biological processes [40]. Secondly, we validated subtype annotations across modalities. For the scATAC-seq data, cell-type labels and gene expression profiles were transferred from scRNA-seq to scATAC-seq data using the addGeneIntegrationMatrix function in ArchR. We then examined the consistency of marker gene expression and activity scores across platforms to ensure robust and reproducible subtype classification.

### 2.4. Accessible Chromatin Peak Calling

Peaks in chromatin accessibility are assumed to mark candidate functional regions of open chromatin [41]. Peak calling was carried out using the addGroupCoverages, addReproduciblePeakSet, and addPeakMatrix functions in ArchR. Specifically, we merged cells within each cell group for the generation of pseudo-bulk replicates and then merged these replicates into a single-insertion coverage file. Next, we obtained insertions from coverage files, call peaks, and merge peaks to acquire a union set of reproducible peaks using the Model-based Analysis of ChIP-seq (MACS2, v2.2.7) [42].

### 2.5. Candidate Cis-Regulatory Element Identification

We linked the constituent peaks to nearby genes using the ArchR addPeak2GeneLinks function. Specifically, cells were combined into overlapping aggregates, and aggregates were annotated based on the predominant cell type. The accessibility of peaks and the expression of nearby genes within 250,000 base pairs were correlated across all aggregates. Highly correlated links between gene expression and region accessibility were identified by addPeak2GeneLinks (corCutOff = 0.4 based on previous studies [43,44]) as candidate *cis*-regulatory elements (CREs) [45,46].

To further define cell-type-specific distal CREs, we used the getMarkerFeatures function in ArchR to identify differentially accessible peaks (marker peaks) for each cell type. Marker peaks were defined using the Wilcoxon test with bias-matching (FDR ≤ 0.05 and Log2FC ≥ 0.57). Finally, the intersection between marker peaks and candidate distal CREs was taken as cell-type-specific distal CREs, reflecting distal regulatory elements that are both significantly open in each cell type and linked to gene expression.

### 2.6. Specificity Analysis of Candidate Cis-Regulatory Elements

To evaluate which genomic regions contribute most to TAM-specific chromatin accessibility, we calculated the τ-index for each CRE. This metric quantifies which CRE is selectively accessible in specific cell types, with higher values indicating greater specificity [47]. For each CRE, we first computed the average chromatin accessibility across cells in each cell type. The resulting values were min–max normalized across cell types to obtain relative accessibility levels. The τ-index was then defined as:

$$\tau_{i}=\frac{\sum_{k=1}^{K}\left(1-P_{i,k}\right)}{K-1}$$

For each CRE i, $P_{i,k}$ represents the normalized accessibility of CRE i in the cell-type k, and K is the total number of cell types. CREs were annotated to four genomic regions, including promoter, distal, intronic, and exonic, based on genomic locations. Statistical comparisons of τ-index across regions were performed using the Kruskal–Wallis test.

### 2.7. Function Enrichment of Cis-Regulatory Elements

The Genomic Regions Enrichment of Annotations Tool (GREAT, v4.0.4) was applied to perform the functional enrichment analysis for *cis*-regulatory elements identified in scATAC-seq [48]. Particularly, differentially accessible distal peaks for each cell type were supplied to GREAT, and the curated GO terms (biological process, BP) were chosen for enrichment with the binomial FDR value.

### 2.8. Motif Enrichment

Motif annotations used to infer transcription factor motif enrichment were derived from the CisBP database (v2.0), and their locations in the genome were identified using motifmatchr (v1.12) [49]. For differential motif enrichment, the total number of identified motifs was computed between differentially enriched peaks. Hypergeometric testing with Benjamin–Hochberg (BH) correction was used to control false discovery rates.

### 2.9. Core Transcription Factor Identification

To uncover upstream regulators driving TAM subpopulation identity, we identified core transcription factors (TFs) with complementary properties, including chromatin binding potential and transcriptional regulatory activity.

For chromatin binding potential, we used chromVAR (v1.12.0) to compute the Pearson correlation between motif deviation z-scores of each TF and its gene expression [50]. The motif z-scores reflect DNA-binding potential in the specific subset. TFs with motif z-scores above the median across TAMs and correlation coefficients >0.5 were considered candidate positive regulators, reflecting TFs with both accessible binding sites and active transcription.

For transcriptional regulatory activity, we applied DoRothEA-based TF activity inference via viper (v1.3.6) on scRNA-seq data, which estimates TF activity based on the expression of curated downstream targets [51]. Core TFs were defined as those identified by both methods, representing high-confidence regulators with coordinated chromatin accessibility and transcriptional impact.

### 2.10. Identification of Prognosis-Associated TAM Subpopulation

Most scRNA datasets lack prognostic data, so we integrated the scRNA-seq data and bulk RNA-seq cohorts with phenotypic information. Specifically, we utilized the prognosis and transcriptomic data from the TCGA-LIHC dataset to identify the subset of TAMs significantly associated with HCC survival using the Scissor algorithm [52]. Overall survival served as the dependent variable using Cox regression. The alpha parameter was set at 0.01. The reliability significance test was conducted using the reliability test function. In addition, the CIBERSORTx deconvolution algorithm was employed to quantitatively compare the difference between the infiltration levels of TAMs and the tumor stage in HCC patients [53]. The scRNA-seq data from this study served as the reference for constructing the signature matrix, and the parameters were maintained at their default settings.

### 2.11. Trajectory Analysis

Trajectories were inferred using Monocle (v3.21) [54]. We manually selected clusters to form a trajectory from Kupffer cells to SPP1^+^ TAMs using the addTrajectory function in ArchR. Cells were ordered based on pseudotime in the trajectory and distributed across 100 equal bins. Across each bin, the average gene expression and TF binding enrichment values were taken and scaled across the trajectory. Integrated pseudotime analyses’ heatmaps of peaks, motifs, and genes were generated using the correlateTrajectories function.

### 2.12. Super-Enhancer Detection and Target Gene Identification

Super-enhancer (SE) regions were identified using the APEC algorithm (v1.2.2), which is specifically designed for scATAC-seq data analysis [36]. To focus on macrophage-specific regulatory elements, we first extracted distal peaks associated with macrophage cells and aggregated the corresponding chromatin accessibility signals from the 500-bp binned TileMatrix. SE calling was performed using the generate.super_enhancer function, with parameters set to super_range = 500,000 and p_cutoff = 0.05, enabling the detection of 500 kb genomic regions significantly enriched for peaks from the same accession, representing coordinated chromatin accessibility. The resulting SE regions were imported into the ArchR project using the addPeakSet function. A peak-by-cell count matrix was then generated for SE regions using the addPeakMatrix function.

To identify SEs specifically active in SPP1^+^ TAMs, we calculated the τ-index for each SE based on the accessibility matrix. SEs with a τ-index > 0.7 and normalized accessibility >0.5 in SPP1^+^ TAMs were defined as SPP1^+^ TAM-specific SEs based on the sensitivity analysis. To assess the biological specificity of the SEs, we performed an external comparison with known SE datasets. Liver tissue-derived SEs were retrieved from the SEdb3.0 database, and genomic overlaps were determined using the bedtools intersect function [55].

To identify putative target genes of SEs, we initially annotated each SE with the nearest gene based on genomic distance. To ensure biological relevance beyond mere proximity, we subsequently integrated matched scRNA-seq data and computed Pearson correlation coefficients between SE accessibility and gene expression levels within SPP1^+^ TAMs. Only genes that exhibited both a coefficient >0.3 and expression levels exceeding the median across all macrophage subsets were retained. This integrative strategy allowed the identification of SE–gene pairs that are not only in close genomic proximity but also display coordinated activity. These SE–gene pairs were used for downstream analyses.

### 2.13. Motif Enrichment Analysis in Super-Enhancer Regions

To identify TFs potentially regulating SEs, we performed motif enrichment analysis on peaks located within SE regions. First, we annotated motifs using the cisBP database for all peaks residing within SE regions, using the addMotifAnnotations function in ArchR. We then applied the getBgdPeaks function to generate background peaks, excluding SE regions, matched for GC content and chromatin accessibility. For each motif, we calculated the observed number of peaks containing the motif located within SE regions and compared it to the expected number under a hypergeometric distribution model. The *p*-values were adjusted using the Benjamini–Hochberg procedure, and motifs with adjusted *p*-values < 0.05 were considered significantly enriched.

### 2.14. Regulatory Network Construction

To elucidate the regulatory landscape of SPP1^+^ TAMs, we constructed a regulatory network comprising TFs, SEs, and target genes that are prognostically relevant. The network was established based on the following components:

(1) Network Nodes: (a) Target gene nodes: Target genes were derived from our super-enhancer analysis pipeline (see Section 2.12), and we performed univariate Cox regression analysis to retain genes associated with poor overall survival (HR > 1, *p* < 0.05). (b) SE nodes: SE nodes correspond to the super-enhancer regions regulating the corresponding prognostic target genes. (c) TF nodes: TF nodes were the core TFs identified in our prior analysis (see Section 2.9).

(2) Network Edges: (a) SE-gene edges were defined by the Pearson correlation between the chromatin accessibility of SEs and the expression level of the corresponding target genes. (b) TF-SE edges were quantified using a linkage score (LS) to represent the regulatory strength between each TF and SE, as described previously [56]. The linkage score between the SE *s* and the TF *t* is calculated as:

$$LS_{s,t}=\sum_{p\in SE_{s}}R_{p,g}^{2}\cdot MS_{p,t}$$

where p is a peak residing within the SE region s, g is the target gene driven by s, $R_{p,g}^{2}$ is the squared Pearson correlation between chromatin accessibility at peak p and the expression level of gene g, and $MS_{p,t}$ is the motif score of the core TF t at peak p. The TF-SE edges with LSs > 10 were kept for network construction.

The final network consisted of undirected edges between SEs and their target genes (weighted by correlation) and between core TFs and SEs (weighted by LS), representing a putative regulatory program in SPP1^+^ TAMs associated with poor prognosis in HCC.

### 2.15. Molecular Docking

The protein sequence of the transcription factor and the DNA sequence of super-enhancers were input into AlphaFold3 to predict the three-dimensional structure of the new complex [57]. Among the generated models, the top-ranked structure (model_0) was selected for further evaluation. The resulting structure was subjected to PDBePISA (https://www.ebi.ac.uk/msd-srv/prot_int/, accessed on 29 May 2025) to assess the binding energy and interfacial area [58].

### 2.16. Drug–Gene Interaction Analysis

The Drug–Gene Interactions Database (DGIdb, www.dgidb.org, accessed on 2 June 2025) is a web resource that provides information on drug–gene interactions and druggable genes from publications, databases, and other web-based sources [59]. We used the DGIdb database to predict drugs that could interact with the SE target genes.

### 2.17. Animal Model and Immunofluorescence Staining

Male C57BL/6 mice (6–8 weeks, n = 3 per group) were randomly assigned to the control group or the HCC group. HCC was established under isoflurane anesthesia (2% induction, 1.5% maintenance) by subcapsular injection of 1 × 10^7^ Hepa1–6 cells in 50 µL of DMEM into the left liver lobe via a small subcostal incision. Mice in the control group underwent sham surgery without cell injection. Post-operative care included buprenorphine analgesia and daily monitoring. Mice were euthanized on day 21. No animals were excluded from the analysis.

Liver tissues were obtained from mice belonging to normal and tumor groups and subsequently fixed in 4% paraformaldehyde for 24 h. After fixation, tissues were embedded in paraffin and sectioned at 3 µm thickness using a microtome. Tissue sections were deparaffinized in xylene and rehydrated through a graded alcohol series. Antigen retrieval was conducted by incubating the sections in 10 mM citrate buffer (pH 6.0) at 98 °C for 10 min. After washing in PBST (1 × PBS with 0.1% Tween-20, Biosharp, Hefei, China), sections were treated with an autofluorescence quencher and blocked with 5% bovine serum albumin for 1 h at room temperature. Following this, sections were incubated overnight at 4 °C with the primary antibodies, including anti-SPP1 (1:200, Proteintech, Wuhan, China) and anti-CD68 (1:200, Boster, Beijing, China). Tissue sections were then incubated with the corresponding secondary antibodies as follows: Alexa Fluor 488-conjugated goat anti-rabbit (1:400, Abcam, Cambridge, UK) and Alexa Fluor 594-conjugated goat anti-rabbit (1:500, Abcam, UK) for 1 h at room temperature. Sections were mounted with a DAPI-containing antifade medium (Sigma-Aldrich, St. Louis, MO, USA). Tissue images were captured using a fluorescence microscope (Nikon, Tokyo, Japan).

### 2.18. Statistical Analysis

All statistical analyses were performed using R software (v4.3.0). Kaplan–Meier curves were used to assess overall survival, and Cox proportional hazards models were applied to estimate hazard ratios with 95% confidence intervals. For group comparisons, the *t*-test or Wilcoxon rank-sum test was used for two groups, and one-way ANOVA or Kruskal–Wallis test for multiple groups, depending on data distribution. All tests were two-sided with a significance threshold of *p* < 0.05.

## 3. Results

### 3.1. Single-Cell Multi-Omics Profiles of HCC Patient Tissues

**Figure 2:**
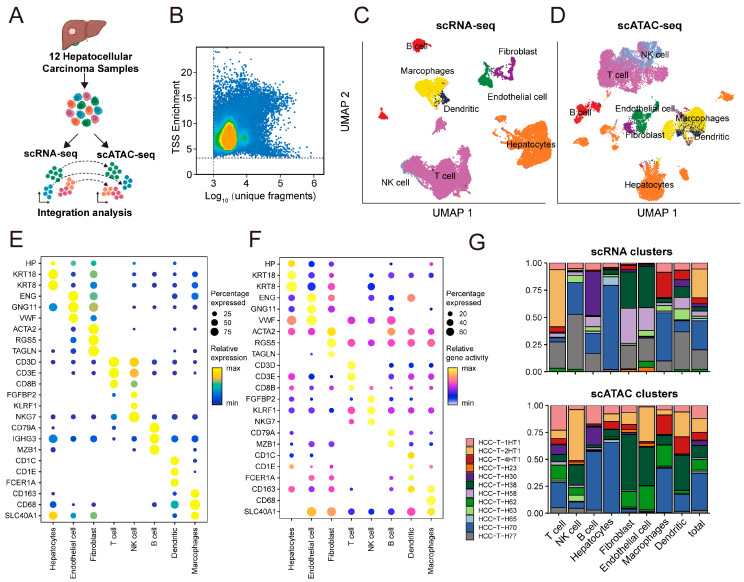
Single-cell multi-omics profiling and annotation of HCC samples. (**A**) Schematic overview of study design. Paired scRNA-seq and scATAC-seq data were obtained from 12 HCC patients for integrative analysis. (**B**) Quality assessment of scATAC-seq data showing transcription start site (TSS) enrichment versus log10-transformed unique fragment counts per cell. Each dot represents single cell, and colors indicate local cell density. (**C**,**D**) UMAP visualization of annotated cell types from scRNA-seq (**C**) and scATAC-seq (**D**) datasets. Each color represents different cell type. (**E**) Dotplot showing the expression of canonical marker genes across cell types identified in scRNA-seq. Dot size represents the percentage of cells expressing gene, and color indicates relative expression level. (**F**) Dotplot showing the chromatin accessibility at corresponding marker gene loci across cell types in scATAC-seq. Dot size represents the percentage of cells with accessible regions, and colors indicate relative gene activity score. (**G**) Bar plots comparing the cell-type compositions across samples for scRNA-seq (**top**) and scATAC-seq (**bottom**). Colors indicate cell-type identity.

To characterize the chromatin accessibility landscape of TAMs in HCC, we retrospectively collected scRNA-seq and scATAC-seq data from 12 patients (Figure 2A). After quality control and filtering of low-quality cells, a total of 90,504 cells were retained for scRNA-seq analysis, and 100,565 cells were retained for scATAC-seq analysis (Figure 2B and Figure S1). For the scRNA-seq dataset, cells were clustered based on transcriptomic similarity and annotated according to canonical marker gene expression. Eight major cell types were identified, including hepatocytes, fibroblasts, endothelial cells, T cells, B cells, NK cells, macrophages, and dendritic cells (Figure 2C). In parallel, cell types in the scATAC-seq dataset were annotated based on chromatin accessibility near known marker genes (Figure 2D), and annotations were further validated using gene activity scores (Figure 2E,F). Next, we quantified the cellular composition in both datasets. All identified cell types derived from scRNA-seq and scATAC-seq were consistently observed across patients (Figure 2G), demonstrating concordance between the transcriptomic and epigenomic profiles.

### 3.2. scATAC-Seq Identifies Major TAM Subtypes in the HCC Tumor Microenvironment

TAMs play a pivotal role in shaping the tumor microenvironment by promoting tumor progression through the induction of inflammation, stimulation of angiogenesis, enhancement of cancer cell proliferation, and suppression of anti-tumor immunity. To delineate the diversity of TAMs in HCC, we first annotated macrophage subpopulations in the scRNA-seq dataset based on the canonical marker gene expression reported in previous studies. A total of nine macrophage subtypes were identified, including SPP1^+^ TAMs, CXCL9^+^ TAMs, SLC40A1^+^ TAMs, TREM2^+^ TAMs, CLEC10A^+^ TAMs, HSP^+^ TAMs, STMN1^+^ TAMs, Kupffer cells, and myeloid-derived suppressor cells (Figure S2A,B).

**Figure 3:**
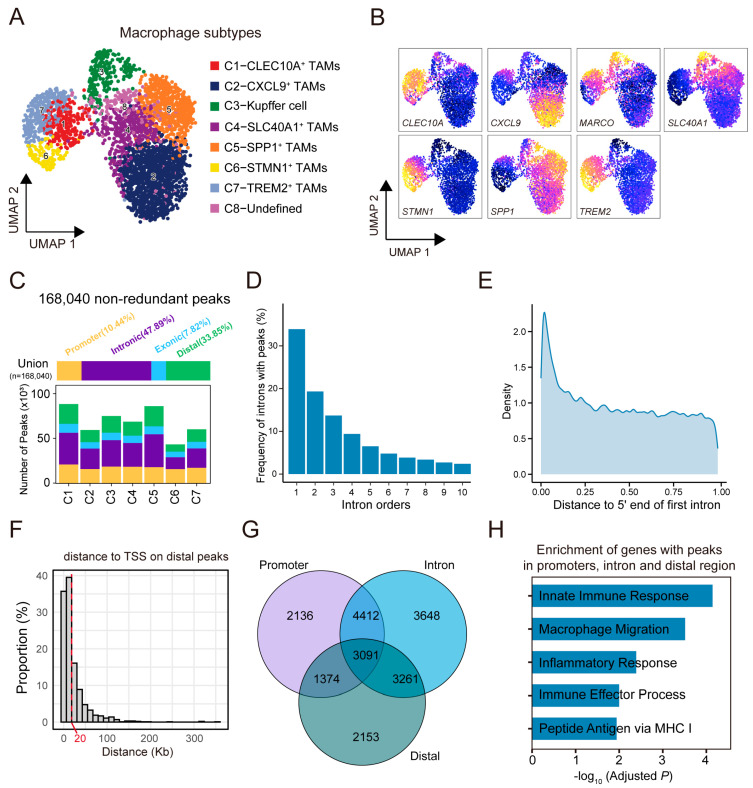
Identification and regulatory characterization of macrophage subtypes in HCC using scATAC-seq. (**A**) UMAP visualization of tumor-associated macrophages (TAMs) identified from scATAC-seq data. Cells are colored by subtype based on label transfer from scRNA-seq, including seven defined subtypes (C1–C7) and undefined clusters (C8). (**B**) Chromatin accessibility at representative marker genes confirms identity of each TAM subtype. Colors represent relative gene activity scores. (**C**) Number of accessible chromatin peaks identified in each TAM, categorized by genomic location. Colors correspond to promoter (red), intronic (purple), distal (green), and exonic (blue). (**D**) Distribution of intronic peaks across intron orders. Y-axis shows percentage of intronic peaks located within each respective intron. (**E**) Density plot showing the distance of intronic peaks to 5′ end of first intron, indicating positional preference near transcription start region. (**F**) Histogram showing distribution of distances from distal peaks to nearest transcription start site (TSS). (**G**) Venn diagram showing overlap of genes associated with accessible peaks in promoter, intronic, and distal regions. (**H**) Functional enrichment analysis of genes with peaks in promoter, intron, and distal regions. X-axis shows -log_10_-transformed and adjusted *p*-values.

Using the label transfer approach, we projected the macrophage subtype annotations from the scRNA-seq data onto the scATAC-seq dataset (Figure S2C–E). We examined the distribution of integration scores across all cells and found that over 90% of cells had scores greater than 0.5, indicating high-confidence alignment. This resulted in the identification of seven major macrophage subtypes: C1 (CLEC10A^+^ TAMs), C2 (CXCL9^+^ TAMs), C3 (Kupffer cells), C4 (SLC40A1^+^ TAMs), C5 (SPP1^+^ TAMs), C6 (STMN1^+^ TAMs), and C7 (TREM2^+^ TAMs) (Figure 3A). The identity of each cluster was further confirmed by the accessibility and activity profiles of the corresponding marker genes (Figure 3B, Tables S2 and S3). A minor population (1.81% of all TAMs) lacked defining marker features and was labeled as undefined, and these cells were excluded from downstream analyses.

To investigate chromatin accessibility in distinct TAM subsets, we identified 168,040 non-redundant regions by merging peaks detected across individual subsets (Figure 3C). Genomic annotation of these peaks reveals a strong positional preference, with intronic and distal intergenic regions accounting for the largest proportions (Figure 3C and Figure S3A,B). Notably, the intronic peaks were predominantly located near the first intron and generally close to transcription start sites (TSSs), suggesting that many of these peaks reside in the 5′ region of the first intron (Figure 3D,E). Similarly, distal peaks were primarily located within 20 kb of nearby TSSs (Figure 3F and Figure S3C).

A total of 3091 genes exhibited accessible chromatin peaks concurrently in promoter, intronic, and distal genome regions (Figure 3G). Functional analysis reveals the significant enrichment involved in immune-related biological processes. These findings suggest that the chromatin regulatory landscape captured by scATAC-seq underlies essential macrophage functions within the microenvironment (Figure 3H).

### 3.3. Cell-Type-Specific Landscape of Cis-Regulatory Elements in TAMs

**Figure 4:**
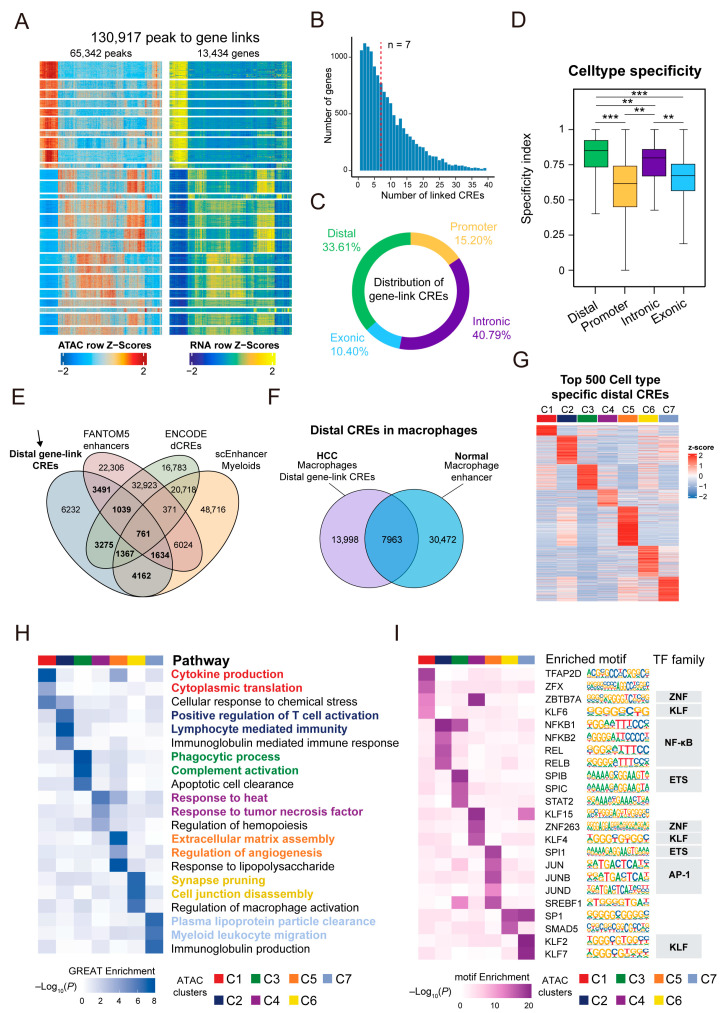
Distal regulatory elements exhibit cell-type specificity and reveal subset-specific transcriptional regulation in TAMs. (**A**) Heatmaps showing peak-to-gene links across candidate *cis*-regulatory elements (CREs) and genes, aligned by ATAC signal (**left**) and gene expression (**right**) across macrophage subtypes. Colors indicate z-score-scaled values per row. (**B**) Distribution of number of linked CREs per gene. (**C**) Genomic annotation of gene-linked CREs. (**D**) Boxplots comparing cell-type specificity index across distal, promoter, intronic, and exonic CREs. Statistical significance was assessed using Wilcoxon rank-sum test. (**E**) Venn diagram displaying overlap of distal gene-linked CREs with known enhancer datasets from FANTOM5, ENCODE, and scEnhancer. (**F**) Comparison of distal CREs in HCC TAMs with annotated enhancers in normal macrophages. (**G**) Heatmap showing z-score normalized accessibility of the top 500 most significant distal CREs across cell types. Each row represents one CRE; each column represents one macrophage subtype. (**H**) GREAT functional enrichment of distal CREs across macrophage subtypes. Color indicates −log_10_(*p*-value) of pathway enrichment. (**I**) Transcription factor motif enrichment in subtype-specific distal CREs. Each tile reflects −log_10_ (*p*-value) enrichment score. TFs are grouped by family. ** *p* < 0.01, *** *p* < 0.001.

The above findings prompted us to further explore the cell-type-specific regulatory landscape of TAM subsets. As *cis*-regulatory elements (CREs) are defined as accessible genomic regions that influence gene expression [45,46], we performed peak-to-gene linkage analysis to associate chromatin accessibility peaks with the expression of nearby genes (within ±250 kb). Peaks showing a significant positive correlation with gene expression (correlation coefficients >0.4) were defined as candidate CREs. As a result, we identified 65,342 putative CREs across TAMs (Figure 4A). These CREs were linked to 13,434 genes, with each gene associated with a median of seven CREs, suggesting complex regulatory interactions between genes and their distal elements (Figure 4B). Notably, a substantial proportion of the CRE-linked genes overlapped with differentially expressed genes across TAM subtypes (Figure S4A), indicating that CREs may contribute to the transcriptional specificity of distinct macrophage states.

Genomic annotation of the linked peaks reveals that the majority were located within intronic regions (40.79%), followed by distal intergenic regions (33.61%), promoters (15.20%), and exons (10.40%) (Figure 4C and Figure S4B). However, when calculating the specificity (τ-index, method 2.6) for each peak, distal elements exhibited the highest degree of cell-type specificity (Figure 4D), indicating the distal regulatory elements in shaping TAMs’ identity. To further investigate the functional relevance of distal CREs, we annotated them against known enhancer databases. Remarkably, 71.62% (15,279 out of 21,961) overlapped with previously defined enhancer elements cataloged by the ENCODE consortium, FANTOM5 project, and scEnhancer database (Figure 4E). Furthermore, a comparison with annotated enhancers in normal human macrophages reveals 13,998 cancer-specific CREs, suggesting that tumor progression and microenvironmental cues drive epigenetic reprogramming in TAMs (Figure 4F).

Since distal CREs exhibited the highest degree of cell-type specificity, we focused on defining differentially accessible distal CREs across clusters. Using the Wilcoxon test and bias correction, we identified a total of 2893 cell-type-specific distal CREs by intersecting differential marker peaks among clusters with candidate distal CREs. To quantify the degree of specificity, we calculated the τ-index for all 2893 distal CREs. As a result, over 80% of these CREs showed τ > 0.5, a threshold commonly used to indicate tissue- or cell-type-specific accessibility patterns [60], confirming their specified activity across cell types (Figure S4C). To better illustrate these patterns, we visualized the top 500 cell-type-specific distal CREs in a heatmap (Figure 4G). The z-score represents the row-wise standardized ATAC signal of each CRE across TAM subsets. These regions displayed distinct accessibility patterns across cell types, indicating their potential roles in establishing cell identity.

To determine the biological relevance of these distal CREs, we performed GREAT analysis across subtypes (Figure 4H). The functional enrichment results reveal distinct biological processes associated with distal CREs across subsets. For example, peaks in cluster C2 (CXCL9^+^ TAMs) were significantly enriched for immune-related biological processes, including the positive regulation of T-cell activation and lymphocyte-mediated immunity, consistent with their pro-inflammatory phenotype. In contrast, peaks in cluster C3 (Kupffer cells) showed enrichment for phagocytic process and complement activation, which are characteristic of liver-resident macrophages. Notably, cluster C5 (SPP1^+^ TAMs) was enriched for terms associated with extracellular matrix (ECM) remodeling and angiogenesis. ECM remodeling refers to the dynamic alteration of the extracellular matrix composition and structure, facilitating tumor invasion and metastasis [61]. Angiogenesis is critical for sustaining tumor growth and creating an immunosuppressive microenvironment, suggesting a potential pro-tumorigenic role for this subset [62].

To identify candidate trans-acting factors driving cell-type-specific distal CREs, we performed TF motif enrichment analysis on distal CREs in each TAM subset (Figure 4I). SPP1^+^ TAMs (Cluster C5) showed significant enrichment for motifs of the ETS family (SPI1), the AP-1 family (JUNB, JUN, and JUND), and SREBF1, all of which are associated with macrophage lineage specification, as well as cholesterol and lipid metabolism [63,64,65]. The CXCL9^+^ TAMs (Cluster C2) showed strong enrichment for motifs of the NF-κB family (REL, RELB, NFKB1, and NFKB2), consistent with their pro-inflammatory transcriptional profile [66]. Kupffer cells (Cluster C3) exhibited preferential enrichment for SPIC, another ETS family member known to regulate tissue-resident macrophage identity [67]. In addition, members of the ZNF (ZBTB7A and ZNF263) and KLF (KLF2, KLF4, and KLF6) families showed selective enrichment in other subsets, suggesting their involvement in macrophage subtype stabilization and metabolic regulation [68,69,70].

Collectively, these findings suggest that highly specific distal CREs, through interactions with distinct transcription factor programs, orchestrate the transcriptional landscape and functional specialization of TAM subsets.

### 3.4. Identification of Prognostically Relevant Subtypes with Divergent Functional Programs

**Figure 5:**
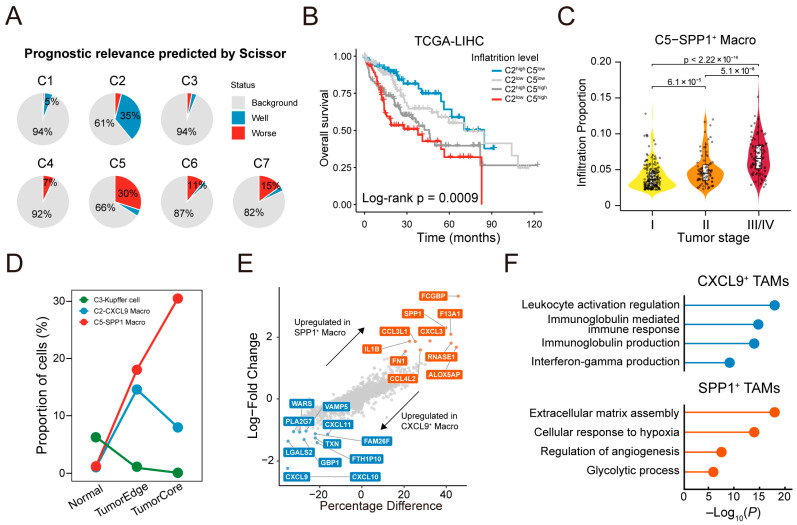
Identification of prognostically relevant TAM subtypes with divergent functional programs. (**A**) Pie charts show predicted prognostic relevance of each macrophage subtype based on Scissor analysis. Cells not assigned to well or worse prognosis were annotated as background cells. (**B**) Kaplan–Meier survival analysis in TCGA-LIHC cohort stratified by inferred infiltration levels of C2 (CXCL9^+^ TAMs) and C5 (SPP1^+^ TAMs) subtypes. (**C**) Violin plots show increasing infiltration of SPP1^+^ TAMs across tumor stages. (**D**) Line plot showing proportion of CXCL9^+^ and SPP1^+^ TAMs across normal adjacent, periphery, and core tumor regions. (**E**) Volcano plot showing differentially expressed genes between CXCL9^+^ and SPP1^+^ TAMs. (**F**) Enrichment of functional pathways based on DEGs in (**E**).

To further elucidate the clinical relevance of macrophage subtypes, we integrated single-cell transcriptomic data with patient survival information to identify subpopulations associated with clinical outcomes. Based on Scissor analysis, the C2 subtype (CXCL9^+^ TAMs) was associated with the highest proportion of favorable prognosis, while the C5 subtype (SPP1^+^ TAMs) correlated with poor clinical outcomes (Figure 5A). Deconvolution analysis further validates these findings. Higher SPP1^+^ TAM infiltration and lower CXCL9^+^ TAM infiltration were significantly associated with worse overall survival (Figure 5B). Additionally, SPP1^+^ TAM abundance was markedly elevated in patients with advanced-stage tumors (Figure 5C), suggesting a role in tumor progression.

To confirm these observations, we analyzed scRNA-seq data from spatially matched peri-tumoral, transitional, and tumor core regions. Macrophages were re-annotated across these regions (Figure S5A,B). KCs (C3 subtype) are tissue-resident macrophages in the liver that self-renew and maintain homeostasis under physiological conditions. We observed a progressive depletion of KCs from non-tumoral regions to tumor cores. Furthermore, CXCL9^+^ TAMs were predominantly enriched in the tumor margin, while SPP1^+^ TAMs displayed consistently increased expression from normal to tumor core regions, suggesting their potential involvement in malignant progression (Figure 5D and Figure S5C).

To explore underlying molecular programs, we performed differential gene expression analysis. *SPP1*, *FN1*, *IL1B*, and *CCL4L2* were significantly upregulated in SPP1^+^ TAMs, while *CXCL9*, *GBP1*, *FAM26F*, and *CXCL11* were enriched in CXCL9^+^ TAMs (Figure 5E and Figure S5D,E). Further functional enrichment analysis reveals that SPP1^+^ TAMs were associated with pro-tumorigenic programs, including extracellular matrix organization, angiogenesis, glycolytic metabolism, and response to hypoxia. In contrast, CXCL9^+^ TAMs were enriched in immune-related pathways, including leukocyte activation, immunoglobulin production, and interferon gamma signaling (Figure 5F). These findings indicate that the transcriptional and functional divergence of SPP1^+^ and CXCL9^+^ TAMs may contribute to their distinct roles in tumor progression.

### 3.5. Microenvironment-Induced Reprogramming of Kupffer Cells into SPP1^+^ TAMs

Given the clinical importance of the SPP1^+^ TAMs, we further explored their functional plasticity within the tumor microenvironment. Recent studies have shown that in inflammatory contexts, liver-resident KCs undergo reprogramming, acquiring features that resemble TAMs [71], suggesting that KCs may shift toward pro-tumorigenic phenotypes in response to the microenvironment. Consistent with this notion, our spatial analysis reveals an opposing infiltration trend of KCs and SPP1^+^ TAMs from normal to tumor core regions, with marked depletion of KCs and accumulation of SPP1^+^ TAMs (Figure 5D), suggesting the possibility of a reprogramming trajectory from KC to SPP1^+^ TAMs during HCC progression.

**Figure 6:**
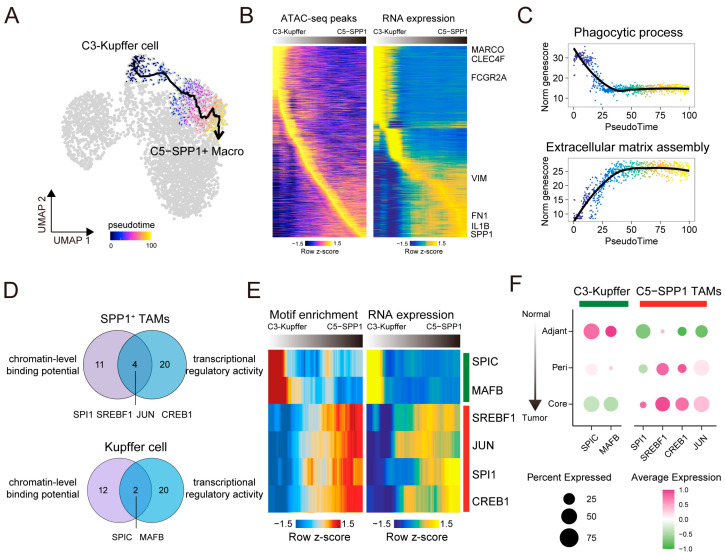
Microenvironment-induced reprogramming of Kupffer cells into SPP1^+^ TAMs. (**A**) Pseudotime trajectory of TAM differentiation inferred from scATAC-seq data. Cells are colored by pseudotime, highlighting a transition from C3 Kupffer cells to C5 SPP1^+^ TAMs. The arrow indicates the inferred direction of pseudotime progression. (**B**) Heatmap showing dynamics of chromatin accessibility (**left**) and gene expression (**right**) along the trajectory. Rows represent top 10% of most variable peaks (**left**) and genes (**right**). Marker genes for Kupffer cells and SPP1^+^ TAMs are labeled. Columns correspond to pseudotime scaled to 0–100 range for visualization. Color scale represents row-wise z-score. (**C**) Pseudotime dynamics of two representative pathways: phagocytic process, enriched in Kupffer cells, and extracellular matrix assembly, associated with SPP1^+^ TAMs. Rows represent scaled pseudotime (0–100), and column shows average gene scores per pathway. (**D**) Venn diagrams showing core transcription factors (TFs) identified using motif enrichment and expression analysis in C3 Kupffer cells and C5 SPP1^+^ TAMs. Overlapping TFs are considered key regulators. (**E**) Heatmap of motifs (**left**) and expression profiles (**right**) of core TFs along trajectory. Rows represent TFs, and columns represent pseudotime bins. Color indicates row-wise z-score. (**F**) Dotplot showing spatial distribution of core TFs from adjacent to tumor region. Dot size indicates percent of cells expressing TFs; color reflects average expression level.

To explore this transition, we constructed a differentiation trajectory from KCs to SPP1^+^ TAMs (Figure 6A). Along this trajectory, chromatin accessibility and gene expression profiles reveal a gradual decline in KC-specific markers (*MARCO*, *CLEC4F*, and *FCGR2A*), while SPP1^+^ TAM-associated markers were progressively upregulated (*SPP1*, *IL1B*, *FN1*, and *VIM*) (Figure 6B). Functional analysis further supports the plausibility of this inferred transition. The canonical phagocytic program of KCs declined markedly, while extracellular matrix assembly pathways associated with SPP1^+^ TAMs were progressively activated during the transition (Figure 6C).

We next sought to identify core TFs that drive the phenotypic transition from KCs to SPP1^+^ TAMs. We focused on TFs that exhibited both chromatin-level binding potential and transcriptional regulatory activity. To this end, we performed motif accessibility and transcriptional activity analyses separately for each subset. In SPP1^+^ TAMs, we identified 15 TFs whose motif accessibility was strongly correlated with gene expression and 24 TFs predicted to actively regulate downstream targets. Four TFs, SPI1, JUN, SREBF1, and CREB1, were consistently supported by both analyses and were defined as core TFs of SPP1^+^ TAMs (Figure 6D, Table S4). Similarly, in Kupffer cells, two TFs, SPIC and MAFB, were identified by both analyses and identified as core TFs (Figure 6D, Table S4).

These core TFs displayed distinct dynamic patterns along pseudotime: *SPIC* and *MAFB* gradually lost both motif accessibility and expression, whereas SREBF1, JUN, SPI1, and CREB1 showed coordinated activation (Figure 6E). Spatial transcriptomics further confirms this divergence. SPIC and MAFB were predominantly expressed in normal and adjacent tissues, while SPP1^+^ TAM-associated TFs (SREBF1, JUN, SPI1, and CREB1) showed peak expression in tumor core regions (Figure 6F). Collectively, these results suggest a potential transcriptional shift coordinated by core TFs, suggesting that the tumor microenvironment may promote the epigenetic and regulatory reprogramming of Kupffer cells toward SPP1^+^ TAMs.

### 3.6. Super-Enhancer Analysis Reveals Potential Druggable Targets in SPP1^+^ TAMs

**Figure 7:**
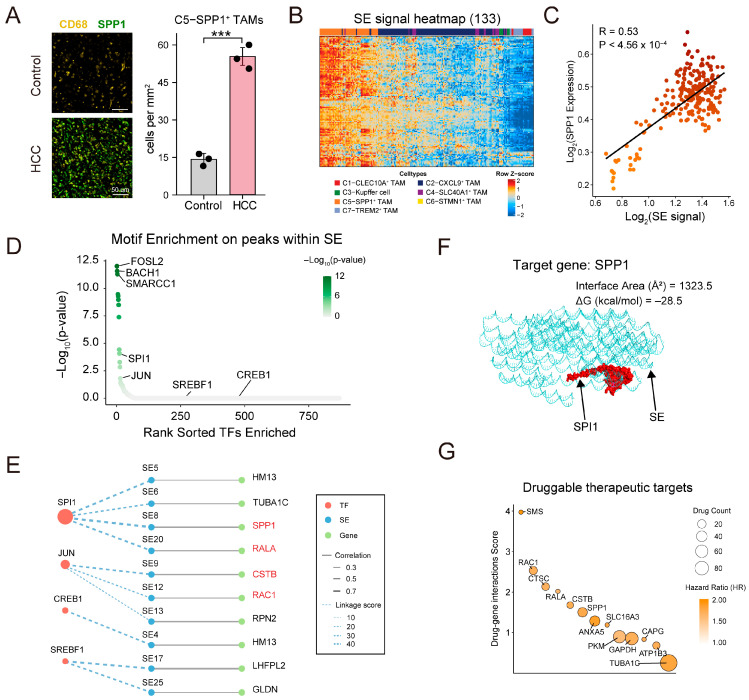
Super-enhancer analysis reveals potential targets in SPP1^+^ TAMs. (**A**) Immunofluorescence staining of CD68 (yellow) and SPP1 (green) in liver tissues from control and HCC mice (n = 3 per group). Quantification shows significant increase in SPP1^+^ TAMs in HCC. Data are shown as mean ± SD; *** *p* < 0.001. (**B**) Heatmap showing normalized signal intensity of 133 super-enhancers (rows) across seven macrophage subtypes (columns), revealing subtype-specific enrichment in SPP1^+^ TAMs. Each row represents one SE region, and each column corresponds to one TAM subtype. (**C**) Correlation between SE signal and *SPP1* expression. R and *p*-values were calculated by Pearson correlation. (**D**) Motif enrichment analysis of TFs on peaks within SE region. The TFs were ranked based on statistical significance using hypergeometric testing. (**E**) TF–SE–target gene regulatory network in SPP1^+^ TAMs. Nodes in red represent TFs, nodes in blue represent SEs, and nodes in green represent SE target genes. Node size of TFs corresponds to the significance of motif enrichment within SE-associated peaks, measured as −log_10_(P) from hypergeometric testing. Gene nodes labeled in red indicate known markers of SPP1^+^ TAMs. Inner edges represent regulatory association between each TF and SE calculated by linking scores. Outer edges reflect the correlation between chromatin accessibility and expression. (**F**) Structural modeling of the SPI1 protein bound to the SE region that regulates *SPP1* gene, as predicted by AlphaFold3 and PDBePISA. (**G**) Drug–gene interaction analysis of SE target genes in (**E**). The y-axis indicates druggability score from DGIdb. Dot size reflects the number of drug interactions, and dot color indicates hazard ratio in HCC.

SPP1^+^ TAMs may represent a pro-tumorigenic subset in HCC. To experimentally validate the computationally predicted enrichment of SPP1^+^ TAMs, we established an orthotopic HCC model in C57BL/6 mice. Liver sections were subjected to immunofluorescence staining for CD68 and SPP1 to identify SPP1^+^ TAMs. Quantitative analysis reveals that SPP1^+^ TAMs were markedly increased in HCC tissues compared to healthy livers, with the proportion rising from 14.78% in control livers to 51.63% in tumors (Figure 7A and Figure S6A). These results support the biological plausibility of our in silico analysis and highlight the pro-tumoral accumulation of SPP1^+^ TAMs in vivo.

Given the strong contribution of distal CREs to cell-type specificity and the central role of super-enhancers (SEs) in controlling cell identity and disease-driving genes, we developed a framework to identify SEs and putative target genes in SPP1^+^ TAMs by combining scATAC-seq and scRNA-seq data (Figure S6B). As a result, we identified 133 SEs specifically active in SPP1^+^ TAMs (Figure 7B and Figure S6C). Given the lack of ChIP-seq data at a single-cell resolution, we sought to validate the identified SEs by comparing them to a known SE database. We downloaded liver-derived SE annotations from the SEdb3.0 database and found that 37 out of 133 SEs (27.8%) overlapped with annotated liver SEs (Table S5). In addition, each SE was linked to its nearest gene and was retained only if it showed positive correlation with gene expression within SPP1^+^ TAMs. We observed that the SE signal was significantly correlated with the expression of the marker gene SPP1 (Figure 7C), suggesting that SEs may regulate the phenotype of SPP1^+^ TAMs in the tumor microenvironment.

To identify candidate trans-acting factors that regulate SE activity, we performed motif enrichment analysis on peaks within SE regions using the cisBP database (Figure 7D). Transcription factors such as FOSL2, BACH1, and SMARCC1 were among the top-ranked hits, suggesting their potential involvement in SE regulation. We focused on four core TFs (SPI1, JUN, SREBF1, and CREB1) previously identified in SPP1^+^ TAMs based on chromatin-level binding potential and transcriptional regulatory activity (Figure 6E and Figure 7D). Among them, SPI1 and JUN exhibited strong motif enrichment within SEs, and SREBF1 and CREB1 exhibited moderate enrichment. These findings support a key regulatory role for these core TFs, particularly SPI1 and JUN, in shaping SE activity in SPP1^+^ TAMs.

Next, we constructed a regulatory network to characterize the molecular program of SPP1^+^ TAMs promoting HCC progression (Figure 7E). The network consists of SEs, SE-regulated target genes, and core TFs. Based on Cox regression analysis, we screened out 31 genes related to poor overall survival from 133 SE target genes (Figure S6D,E, Table S6). Meanwhile, the SEs corresponding to these 31 target genes were also allocated for network construction. The core transcription factors were identified in previous analysis (SPI1, JUN, SREBF1, and CREB1; Figure 6E). We calculated the correlation between SE chromatin accessibility and target gene expression levels to characterize the association between SE and target genes. We also integrated motif enrichment and chromatin–gene information and calculated linkage scores to characterize the connection between TFs and SEs. In this network, SPI1 exhibited the highest connectivity. SPI1 and JUN were linked to multiple SEs associated with marker genes, including *SPP1*, *RAC1*, *RALA*, and *CTSC*, indicating the essential node in the transcriptional control of SPP1^+^ TAMs. To explore the potential of SPI1-SE interactions at the structural level, we used molecular modeling to predict the binding interface between SPI1 and the SE region regulating *SPP1*. The modeled complex reveals a large interface area (1323.5 Å^2^) and favorable binding energy (ΔG = −28.5 kcal/mol), supporting a stable interaction potential (Figure 7F).

To assess the translational potential of SE-driven programs, we queried drug–gene interaction databases (DGIdb) for candidate interventions targeting the 31 poor-prognosis genes. We identified 13 candidate druggable genes, including *SMS*, *RAC1*, *CTSC*, and *SPP1*. These genes exhibited high drug–gene interaction scores and hazard ratios, suggesting promising targets for future therapeutic development aimed at modulating the immunosuppressive function of SPP1^+^ TAMs in HCC (Figure 7G, Table S7).

## 4. Discussion

TAMs are among the most abundant and functionally versatile immune cells in the TME in HCC. Their roles in promoting tumor progression, modulating the immune response, and influencing therapeutic resistance have been well established. However, despite their importance, the regulatory programs underlying TAMs’ phenotypic diversity remain incompletely understood. In this study, we leveraged integrative scRNA-seq and scATAC-seq to provide a comprehensive dissection of TAM subtypes and their epigenetic regulation in HCC. Our findings not only map the chromatin accessibility landscape of TAMs at a single-cell resolution but also identify transcriptional and regulatory networks that define functionally distinct TAM subsets, with translational implications for TAM-targeted therapies.

Our analysis reveals extensive TAM heterogeneity, characterized by multiple macrophage subsets with distinct transcriptional programs and prognostic significance. Among them, we identified two representative subtypes with divergent functional attributes: CXCL9^+^ TAMs, which were enriched in interferon signaling and anti-tumor activity, and SPP1^+^ TAMs, which exhibited pro-tumorigenic characteristics, including enrichment in extracellular matrix remodeling, hypoxia response, and angiogenesis pathways. Spatial and deconvolution analyses reveal that CXCL9^+^ TAMs preferentially localized to the tumor periphery, whereas SPP1^+^ TAMs accumulated in the tumor core and were associated with advanced disease stages and worse overall survival in multiple cohorts. These spatial and prognostic patterns align with previous reports implicating CXCL9 as a marker of immunoactive macrophages and SPP1 as a regulator of tumor progression and immune evasion [72,73].

We performed immunofluorescence staining in an orthotopic mouse HCC model. The observed increase in SPP1^+^ TAMs in tumor-bearing livers reinforces the biological plausibility of computational predictions. Nevertheless, we acknowledge that the use of a murine model for validation imposes inherent limitations, including species-specific differences in immune contexture and macrophage programming. Therefore, it should be interpreted as a supportive observation of spatiotemporal TAM enrichment rather than definitive validation of the molecular mechanisms identified in human samples. Future efforts incorporating spatial transcriptomics or multiplex imaging in patient tissues will be critical to confirm these regulatory trajectories in a clinically relevant setting.

We focused on the regulatory architecture shaping TAM heterogeneity. Through peak-to-gene linkage analysis, we identified over 65,000 putative CREs, including a large proportion of distal regulatory regions. These distal elements, as opposed to promoter-proximal elements, exhibited the highest levels of cell-type specificity and were preferentially enriched in subtypes, such as SPP1^+^ TAMs. This finding underscores the critical role of distal enhancer regions in shaping subtype-specific gene expression programs. The functional enrichment of genes linked to these enhancers reveals immune-related and pro-tumorigenic pathways, highlighting the functional impact of epigenomic remodeling in the TME. Notably, many of the enhancer-linked genes overlapped with differentially expressed genes, reinforcing the validity of our integrative approach.

We inferred a potential developmental trajectory from liver-resident KCs to SPP1^+^ TAMs. Pseudotime analysis and motif dynamics reveal a gradual loss of KC identity, marked by the downregulation of transcription factors such as SPIC and MAFB, accompanied by the acquisition of an immunosuppressive gene program. These findings support the plausibility of a reprogramming process, as evidenced by increasing chromatin accessibility at distal enhancers enriched for SPI1 and JUN motifs, suggesting a potential shift from a tissue-resident to a tumor-educated macrophage state. Importantly, these observations align with previous reports that have proposed a monocyte-independent origin for a fraction of TAMs in HCC and implicate the TME as a potent driver of TAM plasticity [71,74]. Our findings are consistent with a model in which the TME imposes epigenetic pressures that rewire the identity of KCs toward a pro-tumorigenic phenotype. Of course, we recognize that these results are based on computational modeling and spatial association rather than direct lineage tracing. Although the coordinated loss and gain of core transcription factors support the plausibility of this reprogramming process, definitive validation will require future experiments using in vivo fate-mapping systems or in vitro differentiation assays.

Super-enhancers orchestrate cell identity and hijack in cancer to drive oncogenic programs. We identified 133 SEs that were specifically enriched in SPP1^+^ TAMs. These SEs showed strong correlations with key genes involved in immunosuppression and tumor progression, including *SPP1*, *RAC1*, *CTSC*, and *SMS*. Among these, SPI1 exhibited the highest number of SE connections and regulated markers in SPP1^+^ TAMs. SPI1 is a transcription factor known to govern macrophage differentiation and polarization [75,76]. Structural modeling and binding energy calculations supported a strong physical interaction between SPI1 and SE linked to *SPP1*. Together, these data indicate that the SPI1 may represent the essential node in the transcriptional control of SPP1^+^ TAMs.

The functional relevance of these findings was reinforced by integration with clinical and pharmacogenomic datasets. Genes associated with SPP1^+^-specific SEs were significantly enriched in patients with poor prognosis across multiple HCC cohorts. Moreover, by querying the DGIdb, we identified a subset of SE-regulated genes with high druggability scores, including *SMS*, *RAC1*, *CTSC*, and *SPP1*. These genes represent promising therapeutic targets for macrophage-directed interventions aimed at reprogramming immunosuppressive TME. While our identification of these targets offers translational insights, we recognize that these predictions are derived from databases and remain hypothesis-generating. These interactions have not been experimentally validated in TAMs in HCC. Further functional validation, including drug response assays in co-culture or in vivo models, will be necessary to confirm their therapeutic relevance. Unlike conventional immunotherapies that primarily target T cells [77,78], strategies targeting TAM-specific SEs or their upstream regulators, such as SPI1, may offer a novel approach to modulate innate immunity and improve clinical outcomes in HCC patients [79,80].

The study has several limitations that will need to be addressed in future research. First, while pseudotime and chromatin accessibility data suggest a lineage trajectory from Kupffer cells to SPP1^+^ TAMs, definitive lineage tracing experiments, such as fate-mapping models in vivo, are required to validate this conversion. Second, although we demonstrate strong correlations between transcription factor accessibility, motif binding, and target gene expression, functional perturbation studies are needed to establish causality. The knockdown of SPI1, combined with CRISPR interference assays, would be informative in confirming the mechanistic importance of the SE–TF–target network. Third, our study was conducted in a retrospective cohort of HCC patients and mouse models. Prospective validation and functional studies in patient-derived xenograft models or organoids may provide further translational relevance.

## 5. Conclusions

**Figure 8:**
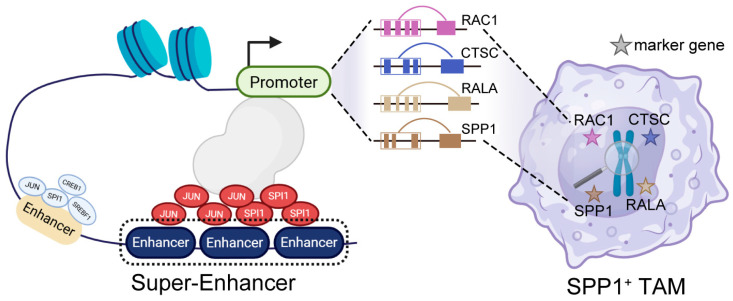
Conceptual model of SPP1^+^ TAM regulation in HCC. Distal CREs within SPP1^+^ TAMs are enriched for motifs of core TFs, including SPI1, JUN, CREB1, and SREBF1. These CREs tend to cluster into super-enhancers, with SPI1 and JUN motifs showing particularly significant enrichment. Cooperative action promotes transcription of marker genes expressed in SPP1^+^ TAMs, such as *RAC1*, *CTSC*, *SPP1*, and *RALA*, contributing to pro-tumorigenic roles of SPP1^+^ TAMs. This model highlights potential therapeutic targets within this regulatory network in HCC.

In summary, our study provides a comprehensive single-cell regulatory map of TAMs in HCC and delineates how chromatin accessibility and transcription factor networks drive macrophage functional heterogeneity (Figure 8). Our findings provide a regulatory basis for macrophage functional heterogeneity in HCC and uncover epigenetic dependencies with translational relevance. These insights may pave the way for novel immunotherapeutic strategies that target the macrophage compartment in liver cancer.

## Data Availability

All datasets analyzed in this study are publicly available. The scATAC-seq data were obtained from the NCBI BioProject under accession number PRJNA944258. The paired scRNA-seq data for the same cohort were retrieved from the Gene Expression Omnibus (GEO) under accession numbers GSE151530 and GSE189903. Additional multi-regional scRNA-seq datasets were obtained from GEO under accession numbers GSE156337, GSE140228, and GSE189903, including liver samples from normal tissue, peri-tumoral areas, and tumor cores. Bulk RNA-seq data were acquired from four HCC cohorts: the TCGA-LIHC cohort from The Cancer Genome Atlas (https://portal.gdc.cancer.gov/), the LIRI-JP cohort from the International Cancer Genome Consortium (ICGC) portal (https://dcc.icgc.org/), and the microarray datasets GSE14520 and GSE116174 from GEO. Code for downstream analyses is available on GitHub (https://github.com/YuGu-CN/TAM_multiomics, accessed on 8 July 2025).

## Supplementary Materials

The following supporting information can be downloaded at: https://www.mdpi.com/article/10.3390/genes16070817/s1, Figure S1: Quality control metrics for scATAC-seq libraries from HCC samples; Figure S2: Integration of scRNA-seq and scATAC-seq data identifies distinct TAM subpopulations in HCC; Figure S3: Genomic annotation and distribution of accessible chromatin peaks across TAM subsets; Figure S4: The *cis*-regulatory elements linking to gene expression across TAM subsets; Figure S5: Spatial distribution and transcriptional regulatory analysis of macrophage subtypes in HCC using multi-regional scRNA-seq data. Figure S6: Identification and clinical relevance of SE-target genes in SPP1^+^ TAMs; Table S1: Summary of datasets used in this study; Table S2: Top 50 differentially expressed genes in each TAM subtype; Table S3: Enrichment analysis in each TAM subtype; Table S4: Predicted core transcription factors regulating SPP1^+^ TAMs and Kupffer cells; Table S5. Overlap of identified SPP1^+^ TAM SEs with known liver SEs from the SEdb3.0 database; Table S6. Univariate Cox regression results for SE-targeted genes in multi-center HCC cohorts; Table S7: Druggable genes associated with super-enhancers in SPP1^+^ TAMs.
