# Supplementary material for: Integrated Single-Cell Analysis Dissects Regulatory Mechanisms Underlying Tumor-Associated Macrophage Plasticity in Hepatocellular Carcinoma
Source: Genes (Basel). 2025 Jul 12;16(7):817. doi: 10.3390/genes16070817 (PMC12294698; doi:10.3390/genes16070817)
Supplement: Supplementary file 1 [file genes-16-00817-s001.zip › Supplementary figures.pdf]

Supplementary Figure

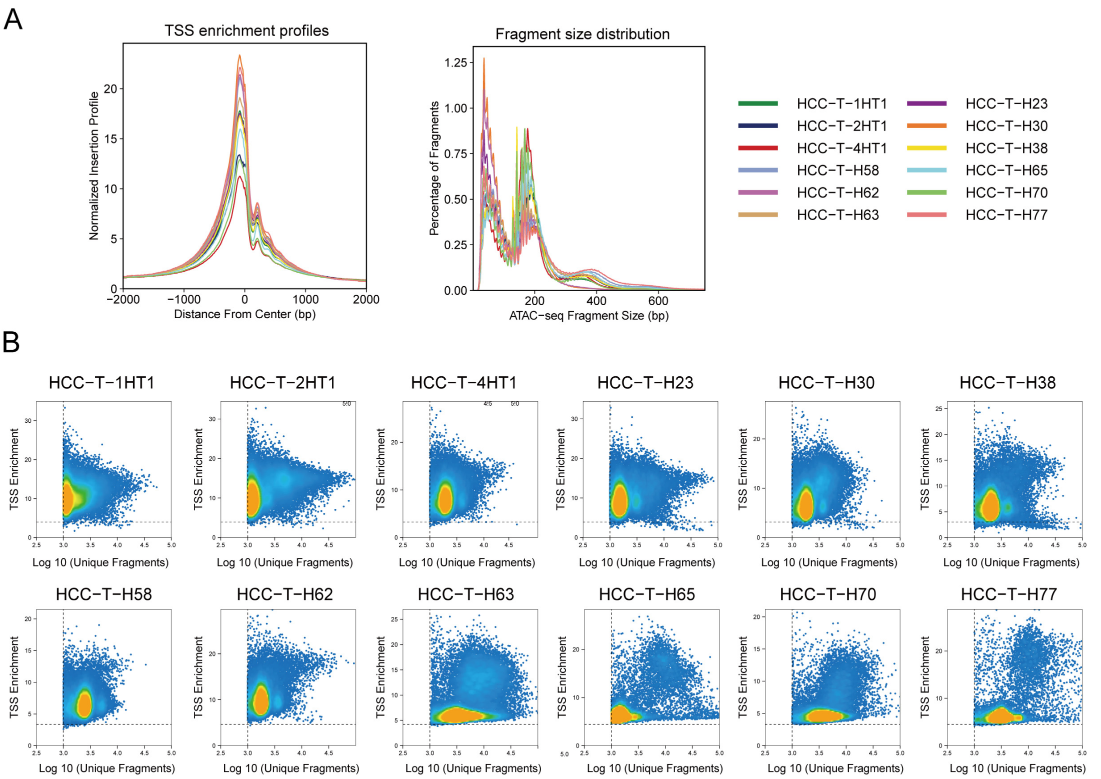

Figure S1. Quality control metrics for scATAC-seq libraries from HCC samples. (A) Transcription start site (TSS) enrichment profiles (left) and ATAC-seq fragment size distributions (right) across all HCC samples. Each line represents one sample (color-coded as indicated). TSS enrichment is plotted as normalized insertion frequency around ±2 kb of the TSS; fragment size is shown in base pairs (bp). (B) Scatter plots showing TSS enrichment versus log$_{10}$-transformed number of unique fragments per cell across all HCC samples. Each dot represents a single cell; color intensity indicates local cell density.

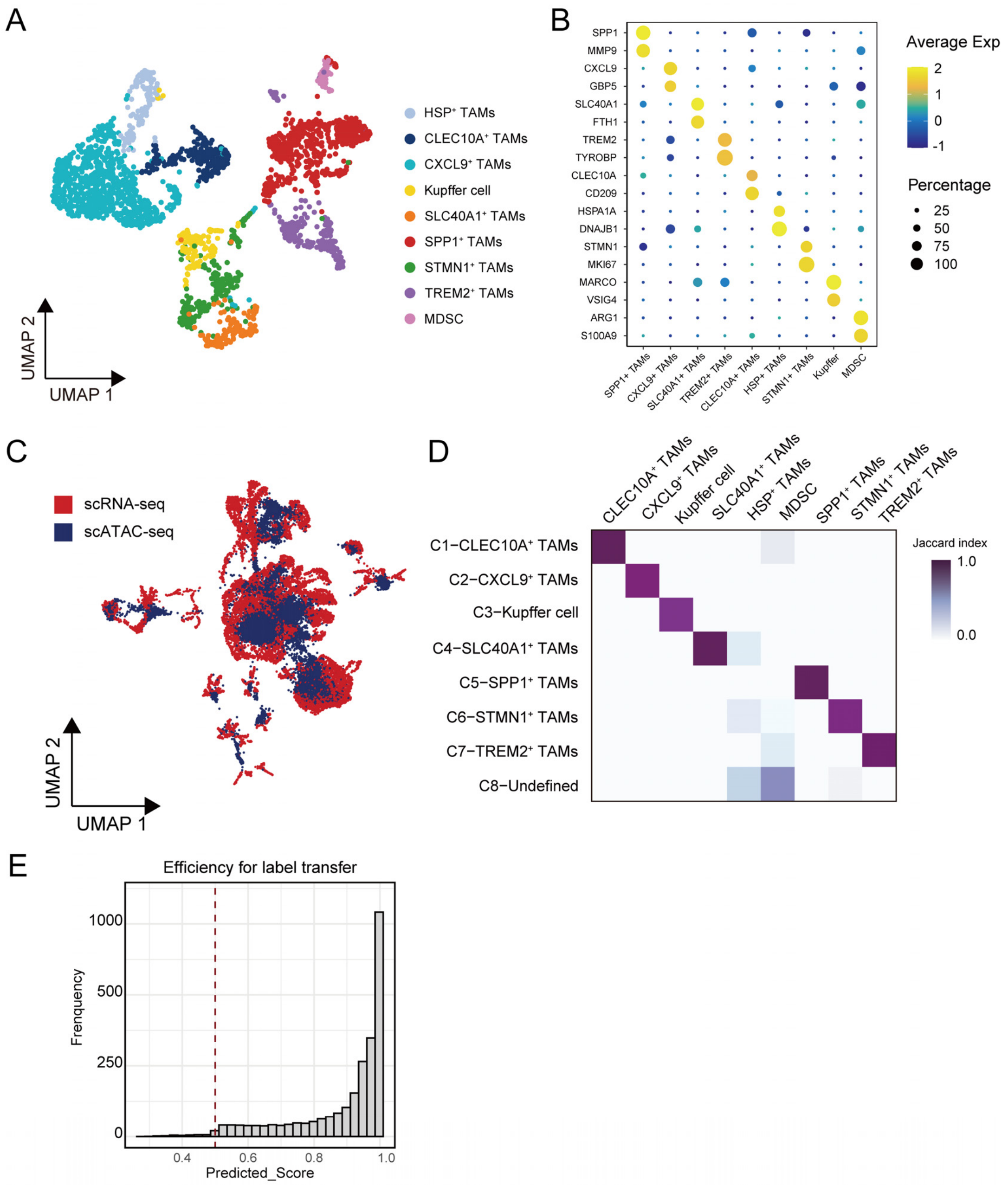

Figure S2. Integration of scRNA-seq and scATAC-seq data identifies distinct TAM subpopulations in HCC. (A) UMAP visualization of single-cell transcriptomes reveals eight distinct macrophage cell types in HCC. (B) Dot plot showing the expression of representative marker genes for each annotated macrophage subpopulation. Dot size represents the percentage of cells expressing each gene, and color represents the average expression level. (C) Joint UMAP embedding of paired scRNA-seq (red) and scATAC-seq (blue) data shows strong alignment of cell populations across modalities. (D) Heatmap of Jaccard index quantifying the overlap between scRNA-seq and scATAC-seq identifying TAMs subsets. (E) Distribution of integration scores from label transfer between scRNA-seq and scATAC-seq in TAMs subsets using ArchR.

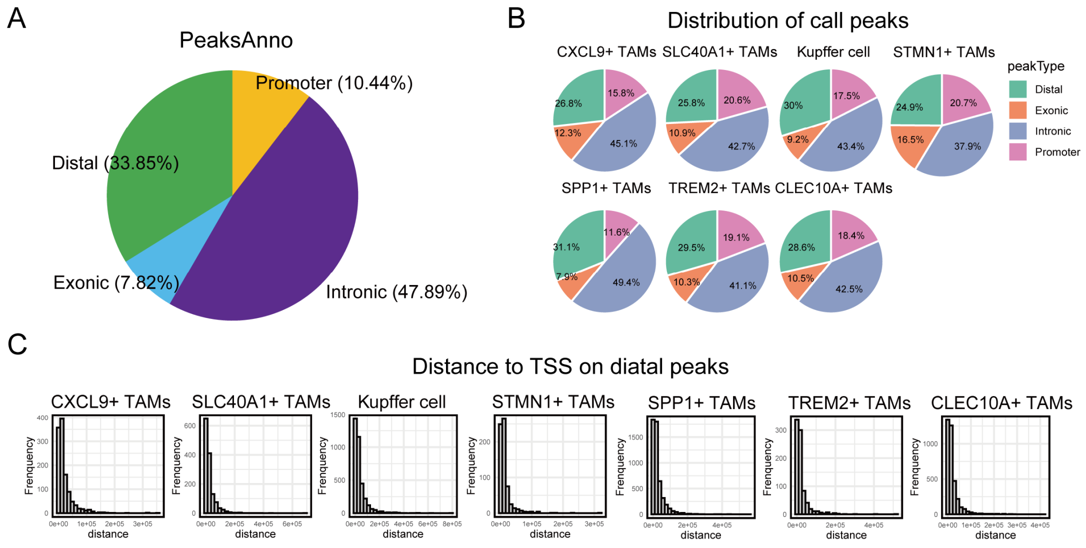

Figure S3. Genomic annotation and distribution of accessible chromatin peaks across TAM subsets. (A) Pie chart showing the genome-wide annotation of all called chromatin peaks in TAMs. Peak categories include promoter (≤1 kb from TSS), intronic, exonic, and distal (>1 kb from TSS) regions. (B) Pie charts showing the relative proportions of each peak type (color-coded) across individual TAM subpopulations. (C) Histograms showing the distribution of distances from distal peaks to the nearest transcription start site (TSS) for each TAM subtype. X-axis represents distance to TSS (in kilobases); Y-axis represents frequency.

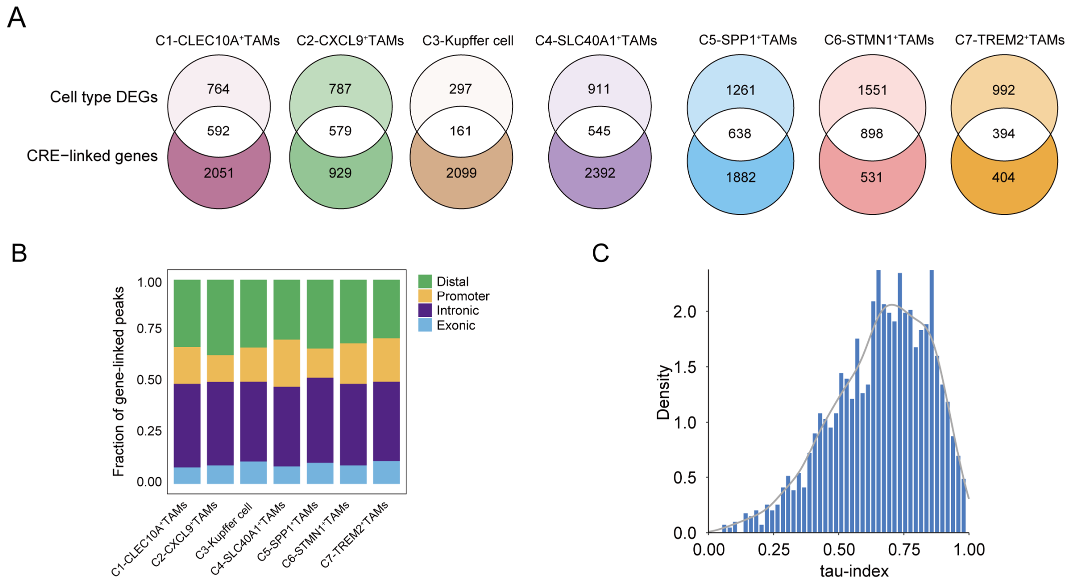

Figure S4. The cis-regulatory elements (CREs) linking to gene expression across TAM subsets. (A) Venn diagrams showing the overlap between cell type–specific differentially expressed genes (DEGs) and genes linked to CREs (CRE-linked genes) in each TAM subtype. Numbers in each segment indicate gene counts. (B) Bar plot showing the fraction of gene-linked peaks annotated as distal, promoter, intronic, or exonic regions

across TAM subsets. Distal and intronic regions account for the majority of CRE-gene associations. (C) Density plot showing the distribution of τ-index values for distal CREs. Higher τ values indicate stronger cell-type specificity. X-axis represents τ-index score; Y-axis indicates density.

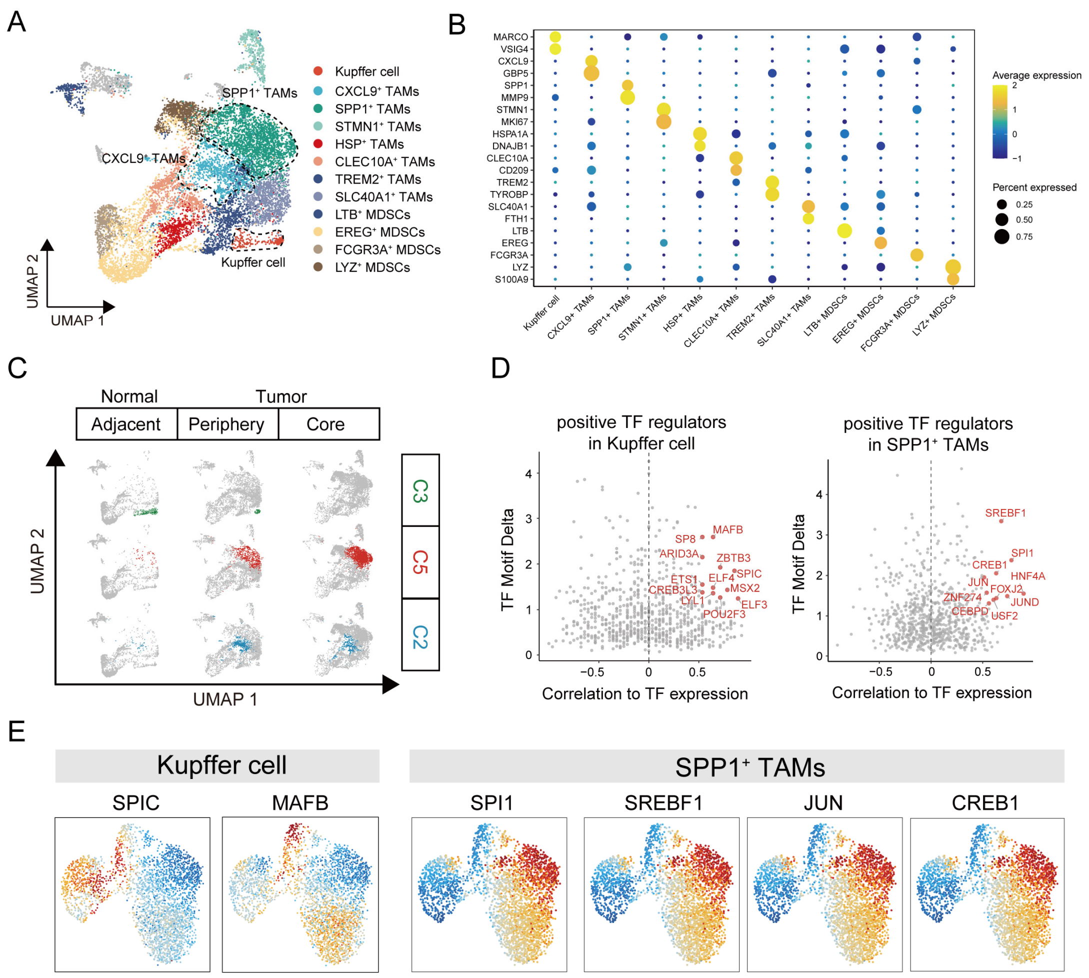

Figure S5. Spatial distribution and transcriptional regulatory analysis of macrophage subtypes in HCC using multi-regional scRNA-seq data. (A) UMAP embedding showing macrophage subsets identified from integrated multi-regional scRNA-seq data. Dashed lines highlight three major subsets (SPP1⁺ TAMs, CXCL9⁺ TAMs, and Kupffer cells) selected for regulatory analysis. (B) Dot plot displaying average expression (color scale) and percent of cells expressing (dot size) for key marker genes across TAM subtypes. (C) Spatial projection of cells from normal adjacent tissue, tumor periphery, and tumor core onto UMAP. C3–Kupffer cells are enriched in adjacent normal regions; C5–SPP1⁺ TAMs dominate tumor core. (D) Scatter plots showing correlation between TF motif accessibility (Motif Δ) and TF expression. TFs with high correlation and motif activity are candidate positive regulators in Kupffer cells (left) and SPP1⁺ TAMs (right). (E) UMAP plots of representative transcription factors showing distinct expression patterns between Kupffer cells and SPP1⁺ TAMs. Color represents normalized expression; scale consistent across panels.

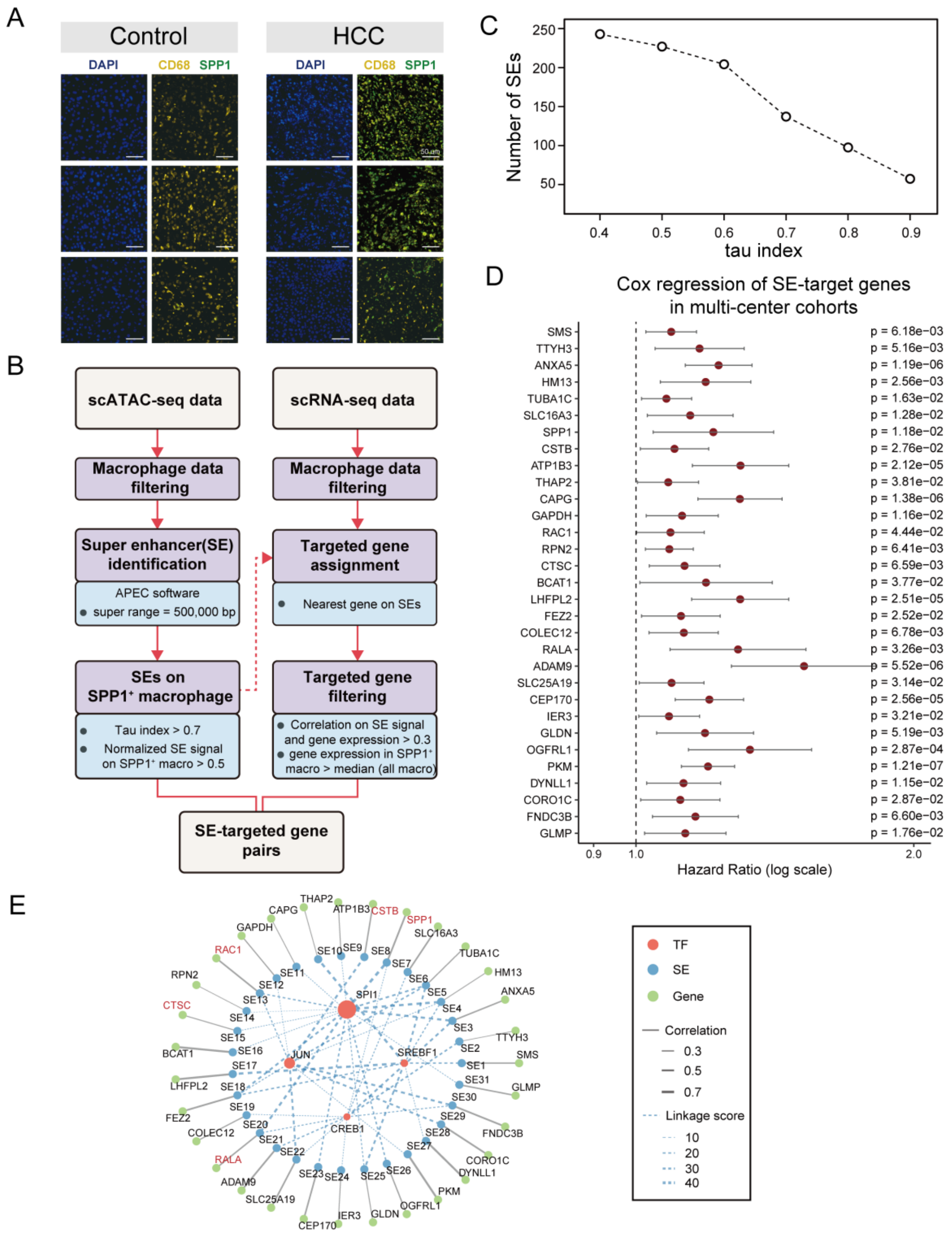

Figure S6. Identification and clinical relevance of SE-target genes in SPP1⁺ TAMs. (A) Representative immunofluorescence staining of CD68 and SPP1 in liver sections from control and HCC mice. Nuclei were stained with DAPI. Scale bar: 100 µm. (B) Workflow for identifying SE-target gene pairs using integrated scATAC-seq and scRNA-seq data. Left: SEs in SPP1⁺ macrophages were defined by APEC software (super enhancer length > 500,000 bp) and filtered by τ-index > 0.7 and normalized SE signal > 0.5. Right: Gene targets were assigned by proximity and filtered by correlation (Pearson r > 0.3) and expression enrichment in SPP1⁺ TAMs (Z-score > 1). (C) Distribution of SEs across τ-index values, illustrating cell type-specificity. (D) Forest plot showing univariate Cox regression results of SE-target genes in multi-center HCC cohorts. Hazard ratios (log scale) with 95% confidence intervals are shown; p-values indicate prognostic significance.

(E) Regulatory network of SE-target genes in $SPP1^+$ TAMs. Nodes represent super-enhancers (blue), target genes (green), and TFs (red). Inner edges denote chromatin linkage scores between TFs and SEs; outer dashed edges represent gene expression-accessibility correlations. Node size of TFs reflects $-\log_{10}(p)$ from motif enrichment. Edge width indicates interaction strength.
